# The Effects of Acupoint Stimulation Combined With Transcutaneous Electrical Nerve Stimulation on Labor Pain: Protocol for a Stepped Wedge Cluster Randomized Controlled Trial

**DOI:** 10.2196/63050

**Published:** 2025-05-26

**Authors:** Yiyun Gu, Xiaojiao Wang, Chunxiang Zhu, Hui Min, Jialu Zhang, Liping Mao, Hua Gao, Hangyun Sun, Xinli Zhu, Chunyi Gu

**Affiliations:** 1 Department of Nursing Obstetrics and Gynecology Hospital of Fudan University Shanghai China; 2 School of Nursing Fudan University Shanghai China; 3 Department of Obstetrics and Neonatology Obstetrics and Gynecology Hospital of Fudan University Shanghai China

**Keywords:** labor pain, transcutaneous electric nerve stimulation, acupressure, complementary therapies, pain management, acupuncture points, medicine, Chinese traditional, pulsed electrotherapy, electrotherapy, nerve, pain, acupuncture, women, protocol, randomized controlled trial, childbirth, pregnancy, pregnant women, quantitative data, qualitative data

## Abstract

**Background:**

Pain experienced during childbirth can significantly impact the progress of labor and the well-being of both the mother and the fetus. Effective management of labor pain is a crucial component of childbirth care. Nonpharmacological methods of pain relief offer notable advantages over pharmacological approaches, including enhanced maternal and fetal safety, equitable access to health care, and greater availability. Among the nonpharmacological options, transcutaneous electrical nerve stimulation (TENS) and acupoint stimulation are two commonly used methods for alleviating pain during labor. However, the clinical efficacy of these methods remains inconsistent, which hinders the generation of high-quality evidence for clinical practice.

**Objective:**

This study aims to assess the effects of acupoint stimulation combined with TENS on labor pain, delivery outcomes, and childbirth experience for women undergoing a trial of labor.

**Methods:**

This is a 12-month stepped wedge cluster randomized trial to be conducted in 4 labor and delivery units (LDUs) at the Obstetrics and Gynecology Hospital of Fudan University. Each unit will implement 4 types of interventions: TENS, acupoint stimulation, TENS combined with acupoint stimulation, and a control group. We aim to recruit approximately 588 pregnant women. The project will be evaluated using both quantitative and qualitative data. Quantitative data will include visual analog scale (VAS) scores, the nonpharmacological to pharmacological pain management interval (NPI), the rate of epidural analgesia, and childbirth outcomes. Qualitative data will include interviews with the women and midwives.

**Results:**

The study commenced on April 1, 2023, and as of March 29, 2024, a total of 600 eligible participants have been enrolled, surpassing the initial target of 588. Data collection has been completed, including quantitative assessments of labor pain intensity, analgesic use, and childbirth outcomes, alongside qualitative interviews with participating women. Currently, data analysis is in progress, with preliminary findings anticipated to be available by March 2025. We hypothesize that TENS combined with acupoint stimulation will demonstrate greater efficacy in managing labor pain compared with standard care. This effect may be observed in key outcome measures, including the VAS score and enhanced maternal childbirth experience.

**Conclusions:**

This study protocol details the interventions of acupoint stimulation and TENS for women undergoing a trial of labor. We introduce a novel outcome indicator termed NPI, which monitors whether the application of nonpharmacological pain relief measures can delay or prevent the use of epidural analgesia. The integration of qualitative and quantitative methods will enrich the research on TENS and acupoint stimulation technology within the realm of nonpharmacological labor pain relief, providing high-quality evidence for the future establishment of industry standards and guidelines.

**Trial Registration:**

Chinese Clinical Trial Registry ChiCTR2300069705; https://tinyurl.com/2s3mkhr7

**International Registered Report Identifier (IRRID):**

DERR1-10.2196/63050

## Introduction

Childbirth pain is widely regarded as one of the most intense forms of pain, often cited by women as the most severe pain they have ever experienced [1]. Approximately 60% of primiparous women report experiencing severe or extremely severe labor pain [2], with 74.2% scoring 8 or higher on the pain perception scale [3]. The intensity of labor pain can lead to anxiety, fear, and tension, which in turn diminishes pain tolerance and exacerbates the cycle of pain. This can negatively affect the childbirth experience, potentially leading to prolonged labor, higher cesarean section rates, and increased risks for both mother and infant [4]. Therefore, effective pain relief strategies are essential for improving maternal health outcomes and the overall childbirth experience.

Pain relief during labor is typically categorized into two approaches: pharmacological and nonpharmacological [5]. Pharmacological methods, including epidural analgesia, nitrous oxide, and intravenous opioids, are generally more effective in managing pain [6]. However, they may have potential drawbacks, such as prolonged labor, dietary restrictions, the risk of intrapartum fever, higher cesarean section rates, and challenges with breastfeeding [7,8]. In contrast, nonpharmacological methods offer a range of alternatives that can reduce pain and anxiety with minimal risk to both the mother and fetus [9]. These approaches, which include transcutaneous electrical nerve stimulation (TENS), aromatherapy, water immersion, and massage, are recommended in clinical guidelines for enhancing the childbirth experience and reducing discomfort [10,11].

TENS and acupoint stimulation have both been extensively explored as nonpharmacological interventions for labor pain relief, each exhibiting distinct mechanisms of action. TENS involves the application of low-frequency electrical currents to stimulate the body’s pain-relief systems, primarily targeting the central nervous system to inhibit pain transmission [12]. Specifically, TENS at frequencies ranging from 2Hz to 100 Hz activates large-diameter Aβ fibers, which, at the dorsal horn level, inhibit incoming nociceptive signals transmitted through small-diameter, slow-conducting Aδ and C fibers that innervate spatially adjacent skin areas. This mechanism produces a pronounced analgesic effect in response to homotopic nociceptive stimulation [13]. TENS has been widely used for relieving chronic pain [14], postoperative pain [15], and cancer-related pain [16]. In the context of labor, several randomized controlled trials (RCTs) have found that TENS can reduce labor pain [17-19], shorten the active labor phase [19], and delay the need for pharmacological analgesia [18]. However, other studies have reported no significant effects on pain relief or labor satisfaction. Consequently, the efficacy of TENS remains inconclusive, with some studies indicating minimal effects on labor satisfaction and pain relief [1,20]. These conflicting results imply that TENS alone may not be adequate for consistent pain management during labor.

Acupoint stimulation, a traditional nonpharmacological method for pain relief rooted in traditional Chinese medicine (TCM), encompasses techniques such as auricular seed acupressure and acupoint massage. This practice is founded on the principle of promoting Qi and blood circulation to alleviate pain [21]. From a contemporary medical standpoint, acupoint stimulation is believed to function through the gate control theory, wherein massage or pressure inhibits nociceptive transmission [22]. In addition, it has been suggested that acupoint stimulation activates Mu opioid receptors, increases serum endorphin levels, and stimulates the release of adrenocorticotropic hormone, all of which contribute to pain relief [23]. Several studies have explored its application during labor, with findings indicating that acupressure can reduce pain during the first stage of labor [21] and that auriculotherapy can alleviate labor pain in primiparous women [24]. However, these studies frequently exhibit methodological limitations, such as small sample sizes and insufficient randomization, which hinder the ability to draw definitive conclusions regarding their effectiveness.

Although both TENS and acupoint stimulation have demonstrated analgesic effects, the existing evidence for nonpharmacological labor pain relief remains limited in strength and lacks standardized implementation protocols, which restricts its clinical applicability [25-27]. The combination of TENS and acupoint stimulation was selected for this study due to the complementary nature of their pain-relieving mechanisms. TENS primarily functions by modulating the nervous system to block pain signals, while acupoint stimulation enhances pain relief by promoting the body’s natural pain modulation processes, such as increasing endorphin release. Together, these approaches may provide synergistic benefits in alleviating labor pain and enhancing the overall childbirth experience. Furthermore, the combination of these two methods may optimize pain relief without increasing risks to the mother or fetus, thus offering a promising alternative to pharmacological interventions.

Based on these considerations, this study aims to evaluate the effects of acupoint stimulation combined with TENS on labor pain, delivery outcomes, and the overall childbirth experience for women undergoing a trial of labor. The primary hypothesis posits that this combination will lead to reduced labor pain, improved delivery outcomes, and a more positive childbirth experience for women. A stepped wedge cluster randomized controlled trial (SWCRCT) design is selected to ensure that all groups will ultimately receive the intervention, thereby promoting equity and adherence to ethical standards. This design also minimizes individual differences by using data from each group’s own participants as a control, while facilitating phased implementation, which effectively addresses resource and personnel feasibility [28].

## Methods

### Trial Design

**Figure 1:**
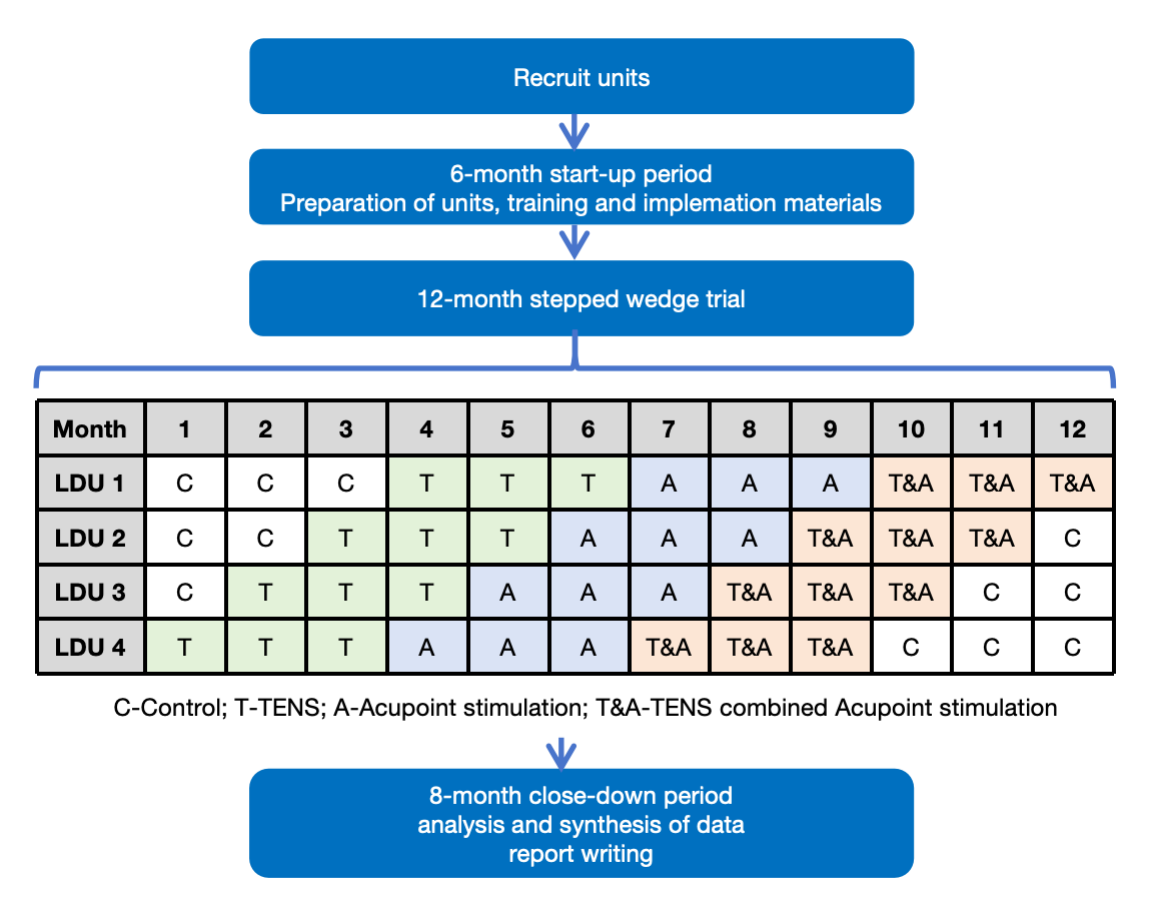
Study flowchart.

This 12-month exploratory SWCRCT will be conducted at the Obstetrics and Gynecology Hospital of Fudan University in Shanghai, China—a specialized tertiary hospital that manages approximately 10,000 births annually across its 2 campuses. The study involves 4 parallel groups, assigned in a 1:1 ratio, each receiving 1 of 4 interventions: TENS, acupoint stimulation, a combination of TENS and acupoint stimulation, or control measures. Randomization is performed at the cluster level using computer-generated sequences to determine the staggered initiation order of interventions across 4 labor and delivery units (LDUs; LDU1, LDU2, LDU3, and LDU4). Each LDU will be assigned to deliver a single intervention for a 3-month period, followed by a transition to the next phase according to the predetermined sequence. This design ensures that each 3-month cohort will comprise unique participants, eliminating crossover exposure to multiple interventions. Participants will be selected based on predefined criteria and confirmed for inclusion before the initiation of the interventions. Figure 1 shows the overall study flow. Recruitment began on April 1, 2023, and all interventions were completed by March 29, 2024, aligning with the 12-month study window. This protocol adheres to the SPIRIT (Standard Protocol Items: Recommendations for Interventional Trials) 2013 checklist [29].

### Eligibility and Recruitment

Participants will be recruited from the Obstetrics and Gynecology Hospital of Fudan University, where they will receive routine antenatal care and plan to give birth. Upon admission to the LDU, trained research staff will identify eligible women through a screening process that includes reviewing medical records and conducting brief assessments. Inclusion criteria require participants to have a singleton pregnancy with cephalic presentation, to be in good maternal and fetal health at admission, to plan to undergo a trial of labor, and to have no cognitive dysfunction or communication difficulties. Women with preexisting or pregnancy-related complications, as well as those with medical indications necessitating a cesarean section, are excluded from the study.

Eligible participants will be approached by trained research staff, who provide detailed information about the study’s purpose, procedures, and potential risks and benefits. Written informed consent will be obtained from all participants before enrollment, and they will be informed of their right to withdraw from the study at any time without consequences.

### Sample Size

The sample size calculation is conducted using the formula for SWCRCTs as outlined by Hemming and Taljaard [30]. The design effect (DE) was calculated based on the meta-analysis by Johnson et al [14], which suggests that a reduction exceeding 0.7 in VAS pain scores indicates a clinically significant effect of TENS. Assuming an expected effect size of 0.7, an intraclass correlation coefficient (ICC) of 0.01, and an estimated sample size of 117 participants in each intervention group, the DE is determined to be 2.16. The total sample size is then estimated using a significance level of 0.05 and a power of 0.8, resulting in 132 participants per group. After accounting for a 10% dropout rate, the required sample size is adjusted to 147 participants per group, culminating in a total of 588 participants. This calculation is verified using PASS 15.0 software (NCSS), which confirms a power of 0.89, thus ensuring the study’s adequacy.

### Randomization and Allocation

This study uses a SWCRCT design, wherein the interventions are allocated at the cluster level, specifically based on the LDUs, rather than at the individual participant level. The randomization process is conducted by a researcher not involved in the intervention using a computer-generated random number list to determine the sequence of interventions across the 4 LDUs. The random allocation sequence is subsequently sealed in opaque envelopes, each corresponding to a specific LDU. This allocation sequence will be disclosed to the midwives in each cluster at the appropriate time. The midwives, trained to implement the intervention, will execute it according to the allocated sequence. This approach ensures allocation concealment and mitigates the risk of selection bias.

### Intervention

A literature analysis method is used to systematically search for relevant studies across multiple databases, including the Cochrane Library, JBI Evidence-Based Nursing Database, Medline, Embase, WanFang Data, SinoMed, CNKI, and VIP. This search uses keywords related to TENS, auricular seed acupressure, and acupressure specifically for women undergoing a trial of labor. A total of 27 studies on TENS, 30 studies on auricular seed acupressure, and 41 studies on acupressure were retrieved. Content analysis is subsequently applied to extract key information from the selected literature. The research team will conduct content extraction, analysis, and synthesis on relevant topics to draft an initial intervention plan for labor pain relief using TENS combined with acupoint stimulation for women in the trial of labor. Based on this draft, expert consultations will be organized to revise the plan, ultimately resulting in the final version of the intervention protocol.

Before the implementation of the project, the midwives at the 4 LDUs will participate in a 1-week training course led by researchers qualified in TCM nursing. The course will cover the operational procedures and assessment of the 3 nonpharmacological pain relief techniques. Competency will be evaluated using a percentage-based scoring system that assesses key aspects such as knowledge of protocols, practical skills, and communication effectiveness. Midwives who achieve a score of 80 or higher will pass the assessment and receive a TCM-based labor pain relief technique certification from the study institution. This certification ensures that all relevant personnel are proficient in the standardized implementation of these techniques before the intervention.

Table 1 shows detailed descriptions of the 3 intervention techniques. These interventions will be administered to participants during labor progression, spanning from the initial onset of contractions to final delivery. All interventions will be individualized according to each participant’s unique labor progression and birthing context while being strictly confined to a single care episode to ensure protocol consistency. To mitigate cross-contamination risks among study groups, designated cluster units (LDU wards) will maintain geographic separation and administrative independence.

Participant adherence to the interventions will be rigorously monitored throughout the study. The intervention protocol will initiate upon the onset of regular contractions and be suspended during intercontraction intervals. During these pauses, midwives will assess maternal comfort levels and dynamically adjust interventions in real time based on participant feedback. For the acupoint stimulation group, adherence will be ensured through direct observation by trained midwives, who will administer massage techniques and maintain continuous stimulation at predefined acupoints. Adherence metrics, including duration, frequency, and intensity of each intervention session, will be recorded using a standardized log sheet. Maternal willingness and comfort will serve as primary adherence indicators, ensuring that interventions remain tolerable and beneficial throughout labor. Participants retain the autonomy to modify or discontinue the intervention at any time, with all adjustments documented for analysis. Regular feedback will be collected through brief interviews and comfort assessments, with findings integrated to refine intervention delivery while safeguarding maternal well-being.

To ensure data accuracy and protocol fidelity, a comprehensive quality assurance framework will be implemented, including (1) regular oversight by the research team, involving scheduled audits to assess adherence to intervention protocols; (2) mandatory use of standardized checklists by midwives to systematically verify and document each procedural step; and (3) prompt documentation and analysis of protocol deviations, with root-cause investigations conducted to address discrepancies. In case of identified nonadherence, targeted retraining sessions will be provided to reinforce proper procedures and ensure understanding of the protocol. These measures are designed to maximize intervention fidelity, ensure methodological rigor, and uphold the integrity of the study.

**Table 1:** Description of intervention protocols.

Intervention types			Detailed descriptions
Low-frequency pulsed electrotherapy or TENS^a^ (T)			Preprocedure preparation: The midwife will prepare the TENS device, electrodes, and connecting wires. The laboring woman will be positioned comfortably, and the skin will be cleansed with warm water. Procedure: Electrodes will be placed on the Hegu (LI4) point, Neiguan (PC6) point, T10-L1, and S2-S4 (Figure 2). The device will be set to alternate between frequencies of 2 Hz and 100 Hz every 6 seconds, with pulse widths of 0.6 ms for 2 Hz and 0.2 ms for 100 Hz. The intensity will be gradually increased from low to high based on the laboring woman's feedback until the optimal stimulation level is achieved. Typically, the intensity for channel 1 (Hegu and Neiguan points) will be set between 6 and 15 mA, while for channel 2 (T10-L1 and S2-S4), it will range from 6 to 40 mA. The midwife will instruct the laboring woman on how to adjust the intensity, empowering her to modify the settings according to her comfort level. Timing of application: TENS therapy will commence at the onset of contractions, with intensity increased during contractions and decreased during periods of relief. This therapy will continue as tolerated until the delivery of the placental. Postprocedure care: Upon completion of the treatment, the device will be turned off, electrodes will be removed, and the skin will be inspected for any signs of irritation. The area will be cleansed to ensure hygiene is maintained.
**Acupoint stimulation (A)**			
	Auricular seed acupressure	Preprocedure preparation: The midwife will prepare a treatment tray containing a kidney dish, 75% alcohol swabs, and vaccaria seeds (Wang-Bu-Liu-Xing) with adhesive tape, tweezers, and a probe. The laboring woman will be positioned comfortably in a sitting, supine, or lateral position to ensure full exposure of the ear. Procedure: The midwife will stabilize the auricle and use a probe to identify the positive reaction points at the bilateral Uterus (TF2), ShenMen (TF4), Endocrine (CO18), Sympathesis (AH6a), and Subcortex (AT4) points (Figure 3). After disinfecting the ear with an alcohol swab, the midwife will apply vaccaria seeds with adhesive tape onto the selected acupoints and press them for 1-2 min per side to enhance stimulation. Timing of application: The procedure will be performed at the onset of contractions. The seeds should be pressed for 1-2 min per contraction or as tolerated until placental delivery. Postprocedure care: The seeds can remain in place for up to 1-2 days post delivery to alleviate uterine contraction pain. If the laboring woman experiences discomfort or requests removal, the procedure should be terminated immediately. After removal, the midwife should clean the auricle, ensure patient comfort, and adjust clothing and bedding.	
	Acupressure	Preprocedure preparation: The midwife should encourage the woman in labor to empty her bladder and bowels, ensure a comfortable position, and maintain warmth. Procedure: During the latent phase of labor, due to the slow cervical dilation, abdominal pain tends to concentrate around the GuanYuan (CV4) point. The midwife can provide a massage using the palm of her right hand in a clockwise direction. Simultaneously, the left thumb can be used to apply pressure to the HeGu (LI4), NeiGuan (PC6), and KunLun (BL60) points (Figure 4). In the active phase of labor, the woman will be advised to adopt a left lateral position with the ZhongJi (RN3) point as the center. Pillows and cushions can be used to support the woman’s head and upper thigh. The midwife will use clockwise abdominal massage and apply pressure to the HeGu (LI4), SanYinJiao (SP6), TaiChong (LR3), and ZuSanLi (ST36) points. For back pain, focusing on the CiLiao (BL32) points, the woman should lean forward against a wall or support herself on the edge of the bed to minimize fetal weight on the spine. The midwife will provide a massage to the lumbar and sacral regions, as well as the back, including the ShenYu (BL23) points, and perform a circular massage around the buttocks with the HuanTiao (GB30) point as the center (Figure 5). Timing of application: Acupoint massage should begin at the onset of contractions and be paused during periods of relief. It may continue as tolerated until placental delivery. Postprocedure care: If the laboring woman experiences discomfort or requests termination, the procedure should be stopped immediately. The midwife should cleanse the skin, adjust the woman’s clothing, and ensure her comfort.	
Combination of TENS and acupoint stimulation (T&A)			Preprocedure preparation: The midwife will prepare all necessary tools, including TENS equipment, Vaccaria seeds, alcohol swabs, tweezers, and a probe. The laboring woman will be positioned comfortably to ensure relaxation. Procedure: Initially, the midwife will apply auricular acupressure and place the TENS electrodes. During contractions, the laboring woman will be encouraged to massage the auricular acupoints, adjust the TENS intensity, and receive an acupoint massage. When experiencing contraction relief, the acupoint massage should be paused, allowing the laboring woman to adjust the TENS settings as needed. This intervention may continue as tolerated until placental delivery. Timing of application: This combined approach may be sustained as tolerated until placental delivery. Postprocedure care: If discomfort occurs, adjustments to the technique should be made, or it should be discontinued. The midwife should turn off and remove the TENS device, inspect the skin, and cleanse the area. Auricular acupressure may be continued for 1-2 days post delivery unless discomfort arises.

^a^TENS: transcutaneous electrical nerve stimulation.

**Figure 2:**
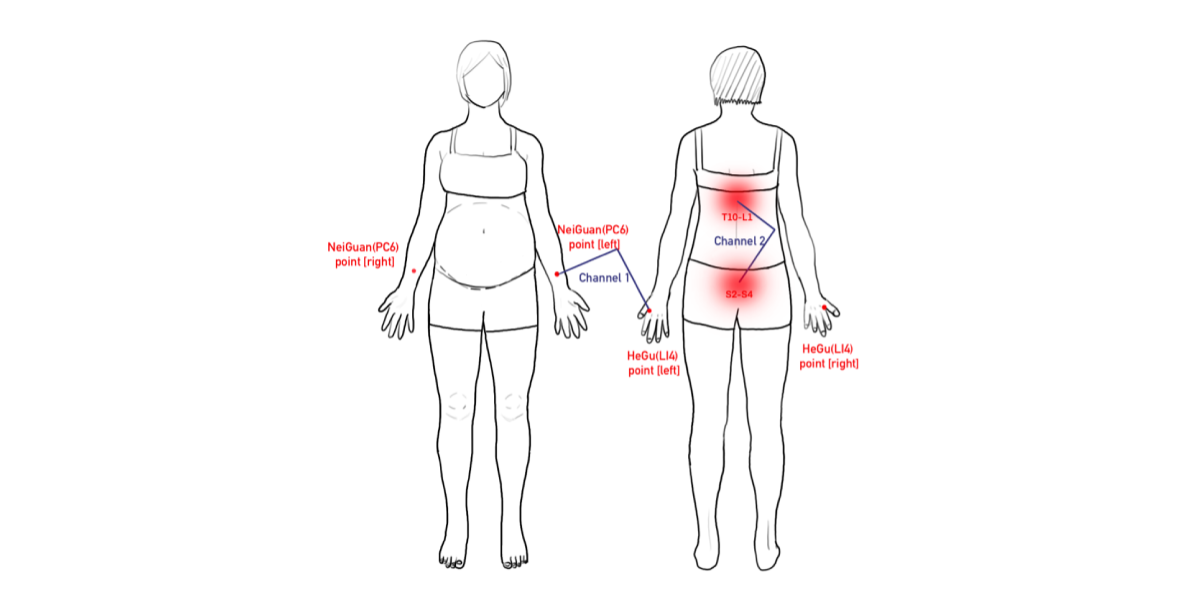
Points for transcutaneous electrical nerve stimulation (TENS) electrodes placed on.

**Figure 3:**
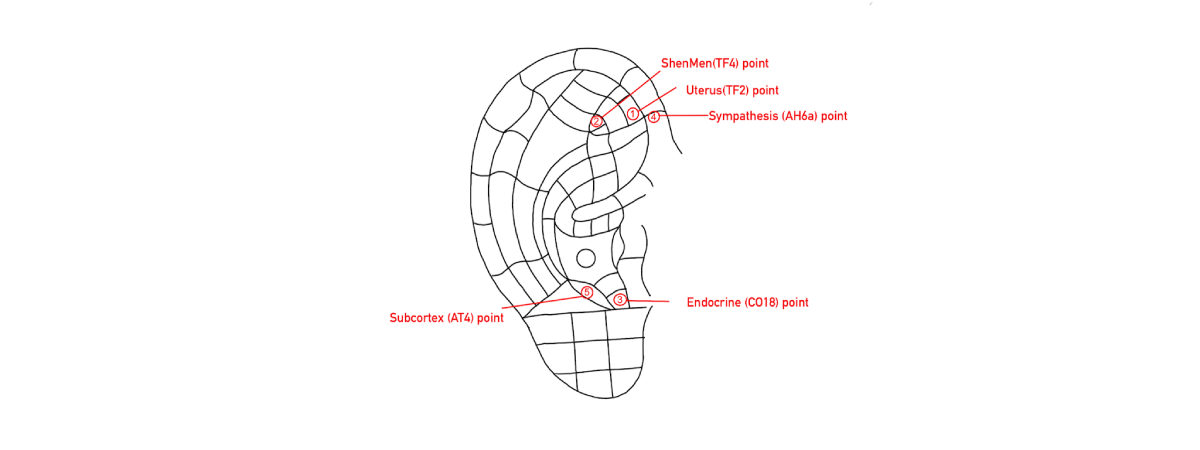
Points for auricular seed acupressure.

**Figure 4:**
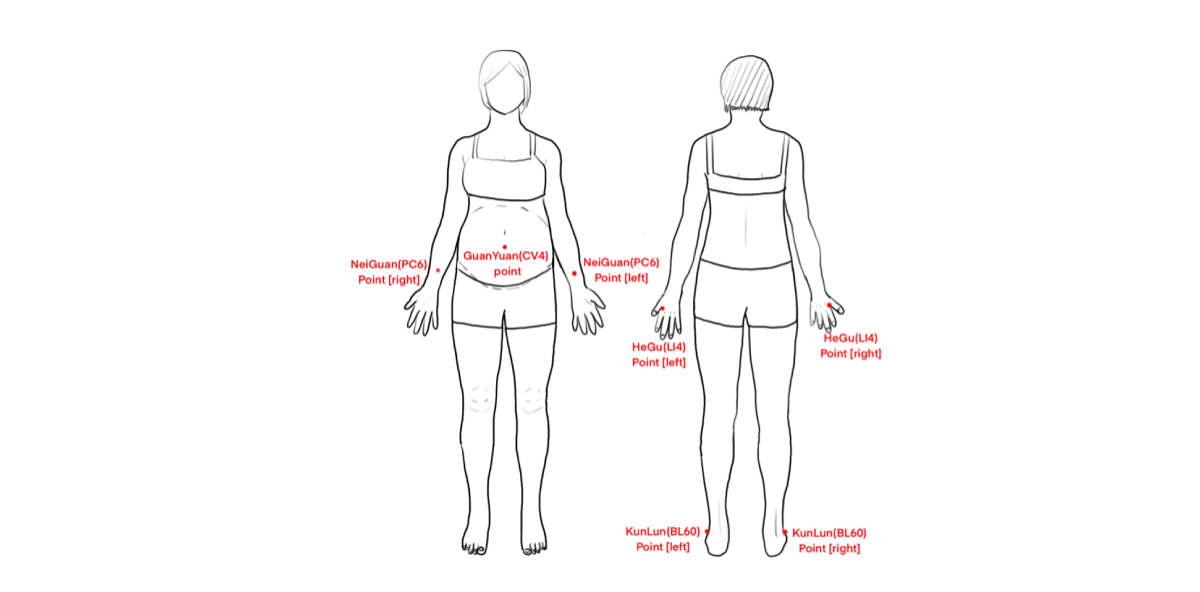
Points for acupressure during the latent phase of labor.

**Figure 5:**
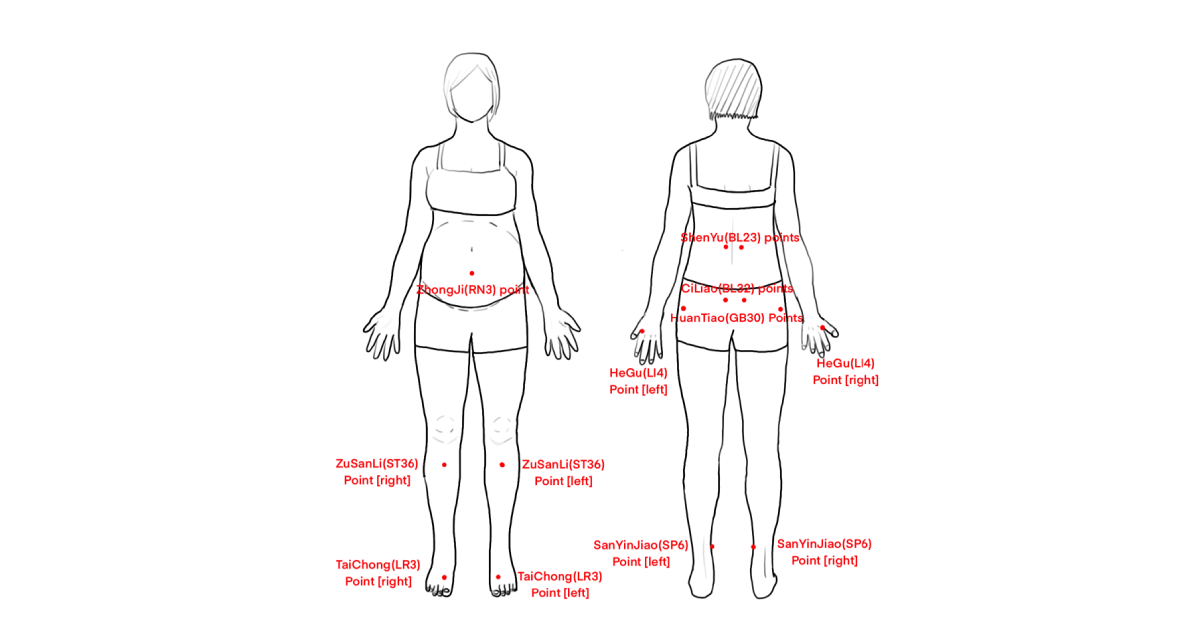
Points for acupressure during the active phase of labor.

### Ethical Considerations

This study has been approved by the Ethics Committee of the Obstetrics and Gynecology Hospital of Fudan University (OGHEC2023-21). The Data Safety Monitoring Committee will conduct continuous safety monitoring of the project implementation and perform annual ethical reviews to ensure procedural coherence. The Data Management and Quality Control Committee will execute quality control evaluations at the initial, intermediate, and preconclusion phases of the project, with a preliminary analysis performed at the midterm stage to guarantee the high-quality execution of the study.

The trial was prospectively registered in the Chinese Clinical Trial Registry on March 23, 2023 (ChiCTR2300069705). All data will be kept confidential, with access restricted to the researchers. Before enrollment, all participants will provide informed consent and will be fully informed of their right to withdraw from the study at any time. Participating women are explicitly assured that their personal data will be managed under strict confidentiality protocols within the research group. All collected study materials will undergo deidentified processing to ensure complete anonymization.

### Blinding

A double-blind design is not feasible in this study due to the necessity for participants to provide informed consent regarding the pain relief methods administered. However, several measures are implemented to minimize potential bias. Midwives delivering the interventions will not be involved in outcome assessment or data analysis, thereby reducing both performance and detection bias. A standardized protocol will be established to ensure consistent delivery of interventions and uniformity in data collection. To minimize measurement bias, outcome assessors will be blinded to the group allocation, and participants will be instructed not to disclose which treatment intervention they receive. Data collectors, who will be independent of the intervention process, do not have access to any information regarding the intervention groups. In addition, statisticians conducting the data analysis will be blinded to group allocation and will not have access to the intervention details. Analyses will be performed according to a prespecified plan to prevent selective reporting. Regular monitoring of adherence to the protocol across the LDUs will be conducted to ensure consistency and reduce the risk of bias. To further enhance the validity and objectivity of the findings, data analysis will be independently verified by a third party to minimize any potential bias.

### Outcome Measures

The primary outcome of this trial is pain perception, measured using the visual analog scale (VAS). Pain perception was assessed from the onset of labor pain, with evaluations recorded every 30 minutes until pharmacological pain relief was administered or delivery occurred. VAS scores ranged from 0 (no pain) to 10 (extreme pain). Secondary outcomes included the interval from the NPI, the rate of epidural analgesia, mode of delivery, the amount of postpartum bleeding within 2 hours, neonatal Apgar scores at 1 and 5 min, labor duration, perineal tear rates, and the overall childbirth experience. The Chinese version of the Childbirth Experience Questionnaire (CEQ) [31] was used to evaluate women’s overall childbirth experience. All secondary outcomes, with the exception of the childbirth experience, assessed post delivery using the CEQ, will be extracted from electronic medical records by the research team, who will not be involved in providing care.

In addition, a purposive sampling method will be used to recruit women from each group for semistructured interviews. The interviews will primarily focus on women’s perceptions and experiences of nonpharmacological pain relief methods during childbirth. The number of women to be interviewed will not be predetermined. Interviews will continue until data saturation is reached, which is the point at which no new information or themes are detected in additional data [32].

### Statistical Analysis

Data will be entered into the database after verification by 2 independent researchers. Quantitative data will be analyzed using SPSS 26.0 statistical software (IBM Corp), following an intention-to-treat (ITT) approach. Normally distributed continuous variables are presented as mean (SD), while non-normally distributed continuous variables are expressed as median (25th percentile-75th percentile). Categorical data are described using frequencies and percentages. Comparisons of normally distributed continuous variables were conducted using ANOVA or the Kruskal-Wallis test, while *t* tests were used for intergroup comparisons. A significance level of *P*<.05 is deemed statistically significant. Missing data will be addressed using multiple imputations.

Qualitative data will be analyzed using NVivo 14 software (Lumivero) through content analysis. Interview materials will be coded and categorized based on key themes derived from the interview guide. Perspectives on similar topics will be grouped and synthesized. Following coding, the software’s classification extraction function will be used to retrieve relevant content from all files under specific codes, allowing for comparison and linkage of different coded content, and identification of primary and secondary viewpoints based on frequency of occurrence for further analysis.

## Results

The study commenced on April 1, 2023, and as of March 29, 2024, a total of 600 eligible participants have been enrolled, surpassing the initial target of 588. Data collection has been completed, including quantitative assessments of labor pain intensity, analgesic use, and childbirth outcomes, alongside qualitative interviews with participating women. Currently, data analysis is in progress, with preliminary findings anticipated to be available by March 2025. We hypothesize that TENS combined with acupoint stimulation will demonstrate greater efficacy in managing labor pain compared with standard care. This effect may be observed in key outcome measures, including the VAS score and enhanced maternal childbirth experience.

## Discussion

### Principal Findings

This study protocol outlines a SWCRCT designed to evaluate the effectiveness of TCM-based TENS and acupoint stimulation for labor pain management. By addressing the limitations of previous research, such as inconsistent implementation techniques and a lack of high-quality evidence [9,33], this study aims to generate robust and clinically relevant findings. We anticipate that the results will provide valuable insights into the potential of nonpharmacological methods to delay the use of pharmacological pain relief, particularly epidural analgesia, while also reducing associated risks such as intrapartum fever [34,35]. Furthermore, the introduction of the NPI as a novel outcome indicator is expected to offer a new perspective on evaluating the efficacy of nonpharmacological interventions. The mixed methods approach, incorporating both quantitative and qualitative data, will provide a comprehensive understanding of the interventions’ impact on labor pain management and women’s childbirth experiences.

### Comparison With Previous Work

This study builds on previous work by conducting a meta-analysis of 32 RCTs evaluating the use of TENS for labor pain relief [33]. The findings indicate that TENS can effectively reduce labor pain; however, the overall quality of the evidence was low, with significant heterogeneity across studies. This variability likely arises from individual differences among laboring women and inconsistencies in the implementation of TENS techniques.

Furthermore, previous studies have predominantly explored comparisons between nonpharmacological pain relief methods and pharmacological pain relief methods [1,36]. However, there is limited evidence on the sequential use of nonpharmacological interventions followed by pharmacological approaches, particularly in the context of delaying the administration of epidural analgesia.

Furthermore, several studies have demonstrated that nonpharmacological pain management can reduce the necessity for epidural analgesia [37] and that epidural analgesia is directly associated with an increased risk of intrapartum fever [34,35]. In light of this, we introduce a novel outcome indicator in this study: the NPI. Compared with the VAS, a subjective evaluation metric for assessing analgesic efficacy, the NPI provides an objective measure to evaluate how nonpharmacological pain management affects the use of epidural analgesia and the incidence of intrapartum fever. This study seeks to evaluate the effectiveness of TCM-based nonpharmacological pain relief methods in delaying and reducing the duration of epidural analgesia, thereby potentially lowering maternal fever rates and improving the quality of labor management.

We have also found that previous studies primarily focused on the effects of nonpharmacological pain relief measures during labor on quantitative health outcomes [19]. In contrast, the perceptions and experiences of women receiving labor pain management have been less thoroughly explored. This study aims to incorporate a mixed methods approach, supplementing the quantitative findings with experiential feedback from various stakeholders. This approach allows for a more comprehensive understanding of the effectiveness of TENS and acupoint stimulation in women undergoing a trial of labor.

### Limitations

However, the study has certain design limitations. First, participants are recruited from a single midwifery institution in Shanghai, which is a tertiary, university-affiliated women’s hospital. Consequently, factors such as geographical location and the level of medical and nursing care may limit the generalizability of our findings to the broader population. Future large-scale multicenter clinical studies will be necessary to further validate the effectiveness of acupoint stimulation and TENS technology for labor pain management in pregnant women from diverse regions and cultural backgrounds. In addition, labor pain intensity in this study was quantified using the VAS score, which carries inherent subjectivity. Finally, participants included in this study are low-risk pregnant women; thus, the applicability of TCM-based nonpharmacological pain relief methods for high-risk women undergoing a trial of labor remains to be further explored and validated.

### Future Directions

This study aims to provide robust evidence supporting the integration of TCM-based nonpharmacological pain relief techniques into routine maternity care. Future research should explore the feasibility of implementing these methods across various health care settings, including community hospitals and primary care centers. Furthermore, additional studies should assess the long-term effects of these pain relief techniques on maternal and neonatal outcomes, as well as their cost-effectiveness. It will also be essential to explore their applicability in high-risk pregnancies and diverse cultural contexts to inform the development of universal guidelines.

### Conclusion

This study protocol details the interventions of acupoint stimulation and TENS for women undergoing a trial of labor. The main objective of this SWCRCT is to determine the effects of acupoint stimulation combined with TENS in reducing labor pain, enhancing women’s childbirth experiences, and improving maternal-fetal health outcomes. In this study, we introduce a novel outcome indicator termed the nonpharmacological to pharmacological pain management interval (NPI), which monitors whether the application of nonpharmacological pain relief measures can delay or prevent the use of epidural analgesia. In addition, the integration of qualitative and quantitative methods will enrich the research on TENS and acupoint stimulation technology within the realm of nonpharmacological labor pain relief, providing high-quality evidence for the future establishment of industry standards and guidelines.

## Abbreviations

CEQ: childbirth experience questionnaire
DE: design effect
ICC: intraclass correlation coefficient
ITT: intention-to-treat
LDU: labor and delivery unit
NPI: nonpharmacological to pharmacological pain management interval
RCT: randomized controlled trial
SPIRIT: Standard Protocol Items: Recommendations for Interventional Trials
SWCRCT: stepped wedge cluster randomized controlled trial
TCM: traditional Chinese medicine
TENS: transcutaneous electrical nerve stimulation
VAS: visual analog scale

## Data Availability

The datasets generated during and/or analyzed during this study are available from the corresponding author on reasonable request.
